# Recurrent Facial Miliaria Crystallina Revealing Underlying Hyperthyroidism: A Report of Two Cases

**DOI:** 10.7759/cureus.117157

**Published:** 2026-09-29

**Authors:** Hajar Dahmani, Ouiame El Jouari, Salim Gallouj

**Affiliations:** 1 Dermatology Department, Faculty of Medicine and Pharmacy, Abdelmalek Essaâdi University, Mohammed VI International University Hospital, Tangier, MAR

**Keywords:** facial miliaria, hyperhidrosis, hyperthyroidism, miliaria crystallina, sweat retention, vesicular rash

## Abstract

Miliaria crystallina is a superficial sweat retention disorder typically associated with fever, heat exposure, or excessive sweating and usually follows a transient course. Recurrent facial involvement without identifiable triggering factors is uncommon. We report two postmenopausal women aged 46 and 54 years presenting with recurrent asymptomatic facial eruptions evolving over several months. Dermatological examination revealed multiple superficial translucent vesicles localized predominantly to the forehead and cheeks. Both patients reported associated heat intolerance and excessive sweating. Laboratory investigations demonstrated suppressed thyroid-stimulating hormone levels with elevated thyroid hormone levels, leading to the diagnosis of hyperthyroidism. Following initiation of antithyroid therapy, the frequency of cutaneous flares progressively decreased, with subsequent cessation of recurrences and improvement in sweating and heat intolerance. Hyperthyroidism-induced hyperhidrosis may contribute to eccrine duct obstruction and promote the development of miliaria crystallina. Recurrent facial miliaria crystallina may represent an unusual cutaneous presentation associated with hyperthyroidism. Recognition of this atypical presentation may facilitate identification and appropriate management of previously unrecognized thyroid dysfunction.

## Introduction

Miliaria is a benign cutaneous eruption resulting from obstruction of the eccrine sweat ducts, leading to secondary sweat retention within the epidermis and/or dermis. Depending on the level of obstruction, several clinical forms have been described. Miliaria crystallina, caused by intracorneal duct obstruction, presents as small, superficial, translucent, and asymptomatic vesicles. Miliaria rubra, secondary to obstruction within the stratum malpighii, is associated with inflammatory erythema and pruritus, whereas miliaria profunda, a rarer form, results from obstruction at or below the dermoepidermal junction [1].

Miliaria crystallina, the most superficial and benign variant, is commonly promoted by heat exposure, high humidity, febrile states, and conditions associated with increased sweating. Certain medications have also been implicated in its development [2].

The condition predominantly affects neonates, while cases in adulthood are rare. Lesions most often involve the trunk and generally follow an acute, self-limiting course, resolving spontaneously within hours to days [3]. Recurrent facial involvement in adults is particularly unusual. To our knowledge, no previous case of recurrent facial miliaria crystallina associated with hyperthyroidism has been reported in the literature. Recognition of recurrent or atypically distributed miliaria crystallina may therefore be clinically relevant, as such presentations can prompt investigation for an underlying condition associated with excessive sweating.

We report two cases of recurrent facial miliaria crystallina, revealing previously unrecognized hyperthyroidism, with no further cutaneous flares observed after initiation of antithyroid therapy.

## Case presentation

Case one

A 46-year-old postmenopausal woman with Fitzpatrick skin phototype III and no significant medical history presented with a recurrent facial eruption evolving over three months, with individual episodes lasting approximately four hours and recurring about four times per week. The lesions resolved spontaneously between episodes, and no clear triggering factor was identified.

Dermatological examination revealed multiple superficial vesicles measuring 1-2 mm in diameter, containing clear fluid, firm on palpation, and arising on otherwise normal-appearing skin, without surrounding erythema or other inflammatory signs. Some vesicles showed a subtle silvery sheen. The lesions were exclusively localized to the face, predominantly involving the forehead and cheeks (Figure 1).

**Figure 1:**
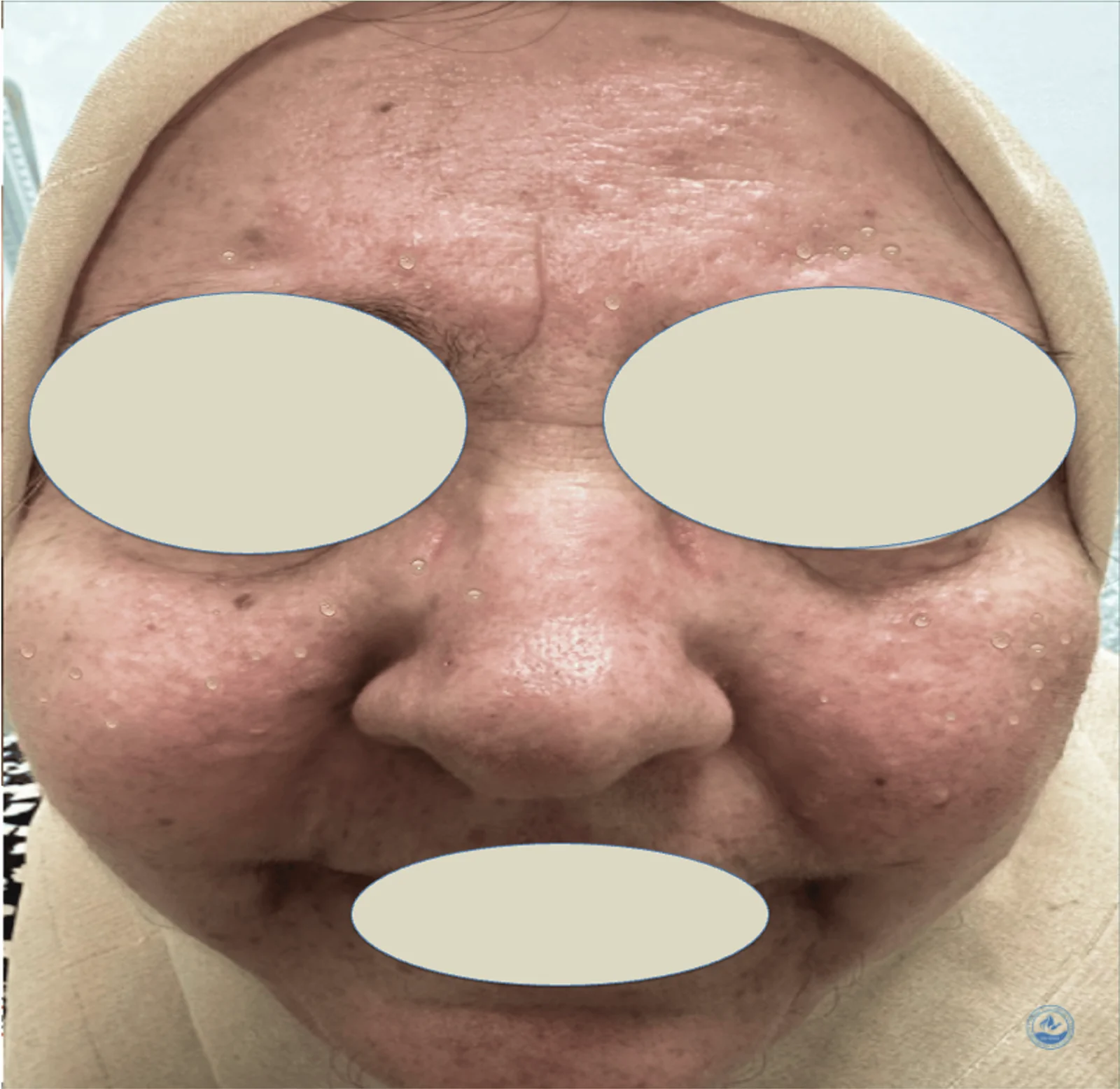
Initial clinical presentation showing multiple persistent superficial translucent vesicles distributed over the forehead and cheeks in a 46-year-old woman

The patient also reported a persistent subjective sensation of heat associated with diffuse hyperhidrosis, without fever. A clinical diagnosis of miliaria crystallina was made, with some features suggestive of the alba variant. Given the characteristic clinical appearance of the lesions, histopathological confirmation was not considered necessary.

In the absence of an obvious environmental or local precipitating factor, a laboratory work-up was performed. Complete blood count, C-reactive protein, and antinuclear antibody testing were within normal limits. Thyroid function tests revealed a decreased thyroid stimulating hormone (TSH) level associated with elevated free thyroxine (FT4) and free triiodothyronine (FT3) levels (Table 1), consistent with overt hyperthyroidism.

**Table 1:** Thyroid function test results in cases one and two TSH: thyroid stimulating hormone; FT3: free triiodothyronine; FT4: free thyroxine.

Thyroid parameter	Case 1	Case 2	Reference range
TSH, mIU/L	0.04	0.10	0.4–4.0
FT4, pmol/L	31.6	28.7	12–22
FT3, pmol/L	15.1	12.2	3.1–6.8

The patient was subsequently referred to the Endocrinology Department, where the diagnosis of hyperthyroidism was confirmed and antithyroid therapy was initiated.

Over the following weeks, the frequency of cutaneous flares progressively decreased. By two months, no further flares were reported, thyroid function had normalized, and there was marked improvement in hyperhidrosis and the subjective sensation of heat.

Case two

A 54-year-old postmenopausal woman with Fitzpatrick skin phototype III and no significant medical history presented with a recurrent facial eruption evolving over five months. Individual episodes lasted at least four hours and recurred approximately once every two days, with spontaneous resolution between flares. No clear triggering factor was identified.

Dermatological examination revealed multiple superficial vesicles measuring 1-2 mm in diameter, containing clear fluid, firm on palpation, and arising on otherwise normal-appearing skin, without surrounding erythema or other inflammatory signs. The lesions were distributed over the cheeks, malar areas, nasolabial folds, and chin, with a few associated milia-like lesions (Figure 2).

**Figure 2:**
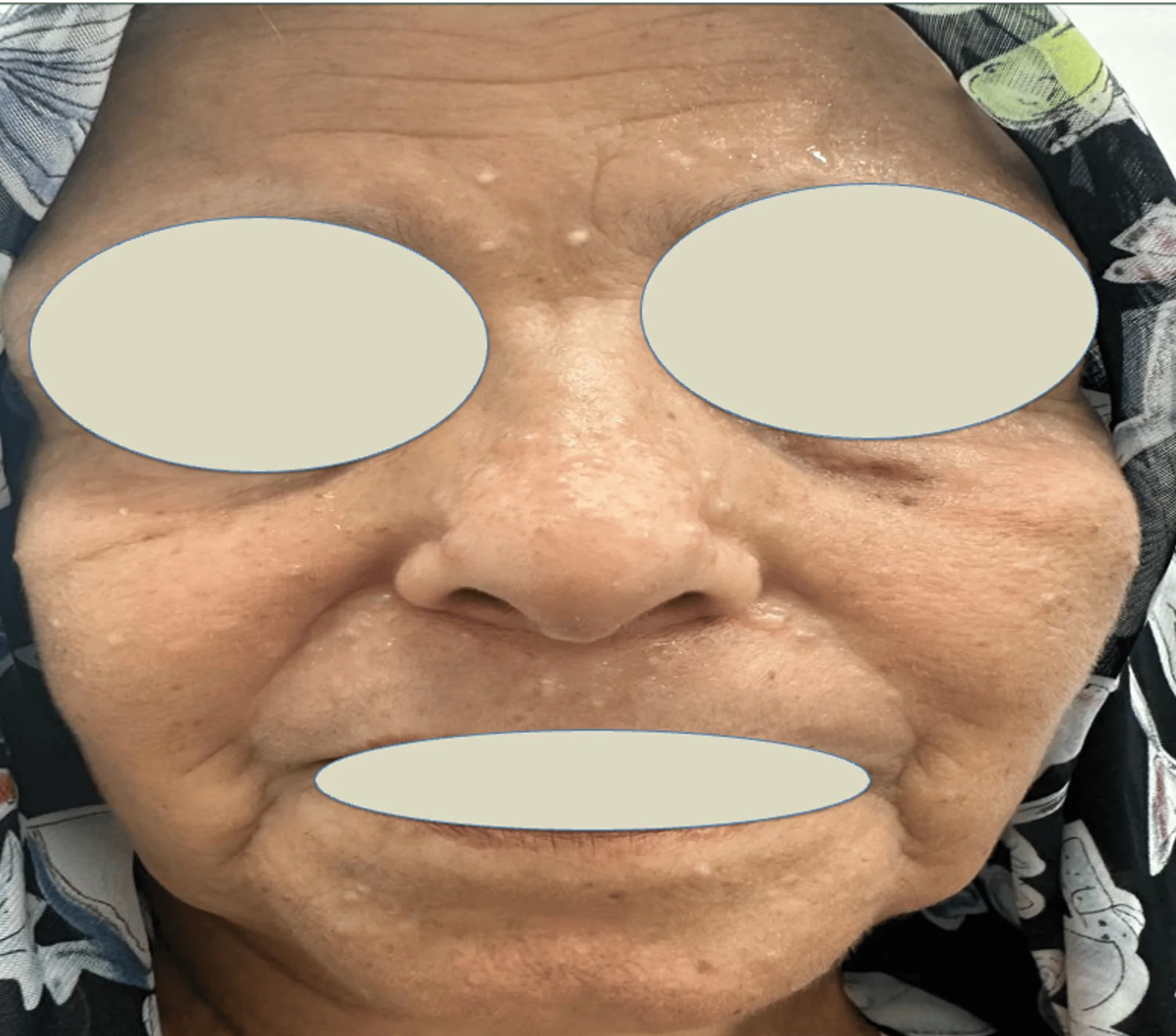
Multiple superficial translucent vesicles distributed over the glabellar region, cheeks, nasolabial folds, and chin, with a few associated milia-like lesions, in a 54-year-old woman

The patient reported a persistent sensation of heat without fever. A clinical diagnosis of miliaria crystallina was made.

In the absence of an obvious environmental or local precipitating factor, a laboratory work-up revealed a decreased TSH level associated with elevated FT4 and FT3 levels (Table 1), consistent with overt hyperthyroidism. The patient was referred for endocrinological assessment and started on appropriate antithyroid therapy.

Following initiation of treatment, the lesions progressively resolved. By four months, complete clearance had been achieved, with no further cutaneous flares observed during follow-up, alongside normalization of thyroid function. No additional dermatological treatment was required.

## Discussion

Miliaria comprises a group of disorders of the eccrine sweat ducts caused by obstruction of sweat flow at different levels of the skin. According to the anatomical level of obstruction, three main clinical forms are recognized: miliaria crystallina, miliaria rubra, and miliaria profunda. In miliaria crystallina, the obstruction is located superficially within the stratum corneum; in miliaria rubra, it occurs deeper within the Malpighian layer of the epidermis; whereas in miliaria profunda, the obstruction is situated at or below the dermoepidermal junction [1].

Miliaria crystallina, also known as sudamina, represents the most superficial form of miliaria. Its pathophysiological mechanism involves occlusion of the terminal eccrine sweat duct at the level of the acrosyringium, which impairs normal sweat excretion and leads to superficial accumulation of sweat with subsequent vesicle formation [1].

Clinically, it is characterized by multiple clear, fragile, non-inflammatory vesicles measuring approximately 1-3 mm and arising on otherwise normal-appearing skin. These lesions are classically described as resembling droplets of water. They are usually asymptomatic and typically resolve spontaneously within hours, sometimes leaving a fine superficial desquamation [1].

Dermoscopic findings in miliaria crystallina are sparsely reported and appear to be nonspecific, with transparent roundish structures corresponding to the superficial vesicles. The diagnosis is usually clinical, based on the characteristic appearance of the lesions, and histopathological examination is generally unnecessary. When performed, histopathology typically reveals an intracorneal or subcorneal vesicle arising from the acrosyringium of the eccrine sweat duct, sometimes accompanied by a sparse neutrophilic infiltrate [1].

The main differential diagnoses of a facial vesicular eruption include herpes simplex virus infection, bullous impetigo, acute contact dermatitis, and non-inflammatory lesions such as eccrine hidrocystomas and syringomas. In our patients, the absence of pain, pruritus, erythema, crusting, grouped vesicles, and any history of recent topical product use made infectious and eczematous causes unlikely. Eccrine hidrocystomas and syringomas were considered unlikely because these lesions are typically persistent, unlike the fragile vesicles in our patients, which ruptured easily and resolved spontaneously. The characteristic superficial, translucent, dew-drop-like appearance supported the diagnosis of miliaria crystallina [1].

As miliaria crystallina is a benign and self-limiting condition, its management is generally conservative and does not require specific pharmacological treatment. Measures aimed at reducing sweating, including maintaining a cool environment and avoiding excessive heat or occlusion, are usually sufficient. When an underlying precipitating factor is identified, its correction is important, particularly in recurrent cases, as persistent triggers may promote further episodes [1].

Miliaria crystallina predominantly affects neonates, particularly in hot, humid, and tropical environments. In this age group, immaturity of the eccrine sweat ducts is thought to facilitate superficial ductal obstruction, while heat and profuse sweating may promote maceration of the stratum corneum and blockage of the eccrine ducts. Rare congenital presentations of miliaria crystallina have also been reported [4].

In adults, miliaria crystallina is rare and is usually reported in association with secondary precipitating factors, particularly excessive sweating, febrile states, heat exposure, occlusive conditions, and prolonged hospitalization [3]. Drug-induced cases have also been described, including those associated with isotretinoin, bethanechol, doxorubicin, and the combination of idarubicin with all-trans-retinoic acid, suggesting that pharmacological interference with sweat production, excretion, or eccrine duct integrity may contribute to lesion development [2,5-7]. Adult cases have also been reported in hospitalized patients with systemic or neoplastic conditions, including glioblastoma [8].

Regarding distribution, lesions in adults most commonly involve the trunk and limbs. More extensive presentations have occasionally been reported, including generalized forms with diffuse vesicles involving large areas of the body [9].

In our two patients, no usual precipitating factor was identified, whereas both reported a persistent sensation of heat and were subsequently diagnosed with overt hyperthyroidism. Hyperthyroidism is associated with increased thermogenesis and excessive sweating, which may favor eccrine sweat duct obstruction and thereby contribute to the development of miliaria crystallina [10]. Although the temporal relationship between antithyroid treatment and resolution of the cutaneous eruptions supports a possible association, causality cannot be established from these two cases alone. Nevertheless, the absence of common precipitating factors, the presence of heat intolerance and hyperhidrosis, and the disappearance of the eruptions following control of hyperthyroidism support thyroid dysfunction as a plausible contributing factor.

To the best of our knowledge, recurrent facial miliaria crystallina associated with hyperthyroidism has not previously been reported. However, a relationship between thyroid dysfunction and other forms of miliaria has occasionally been described. Of note, a granulomatous variant of giant centrifugal miliaria profunda has been described in an infant with congenital hypothyroidism, in whom alterations in eccrine function, sweat electrolyte composition, and keratinization were proposed as contributing mechanisms. Although this represents a different form of miliaria and an opposite thyroid dysfunction, it supports the potential influence of thyroid abnormalities on eccrine sweat duct homeostasis [11].

Moreover, the strictly facial localization in both patients is particularly unusual. One possible contributing factor may be postmenopausal craniofacial hyperhidrosis, which has been described idiopathically in postmenopausal women by Eustace and Wilson [12]. Increased sweating in the craniofacial region could theoretically promote local eccrine duct obstruction, providing a plausible explanation for the exclusive facial distribution observed in our cases and further underscoring the unusual nature of this presentation.

## Conclusions

Although miliaria crystallina is usually a benign and transient eruption, recurrent facial involvement in adults is particularly uncommon and, in the absence of an evident precipitating factor, may warrant investigation for an underlying condition. In our patients, the temporal association between antithyroid therapy and cessation of cutaneous flares raises the possibility of a pathophysiological link with hyperthyroidism. However, this association remains hypothesis-generating, and causality cannot be established from these two cases alone. Recognition of this unusual presentation may nevertheless help identify previously unrecognized hyperthyroidism and support appropriate clinical management.
